# Shedding light on receptor kinase processing

**DOI:** 10.1371/journal.ppat.1013648

**Published:** 2025-11-03

**Authors:** Anna Bannmüller, Adithya Acharya, Martina K. Ried-Lasi, Mariana Schuster

**Affiliations:** 1 Leibniz Institute of Plant Biochemistry, Symbiosis Signalling Group, Department of Molecular Signal Processing, Halle (Saale), Germany; 2 Leibniz Institute of Plant Biochemistry, Receptor Biochemistry Group, Halle (Saale), Germany; University of Tübingen: Eberhard Karls Universitat Tubingen, GERMANY

## How do plants sense their environment?

Plant cells constantly monitor both their surroundings and internal state, enabling dynamic responses to developmental and environmental cues. Central to this sensing capacity are membrane-bound receptor kinases (RKs), one of the largest and most functionally diverse protein families in plants. RKs detect a broad spectrum of signals, including microbial molecules, endogenous ligands, and changes in cellular status. They share a conserved modular architecture: an extracellular domain for signal perception, a single transmembrane domain, and a conserved cytoplasmic kinase domain that initiates intracellular signalling. Although structurally analogous to their animal counterparts, plant RKs evolved independently [1,2]. Their functional diversity stems primarily from their extracellular domains, with over 20 distinct structural classes described to date [1–3]. The repertoire of RKs expressed in a given cell type varies, as some RKs are ubiquitously expressed across tissues, while others are restricted to specific organs or developmental contexts [4].

## How do RKs initiate signalling and how are they regulated?

RK activation is a dynamic, multi-step process that ensures precise cellular adaptation to environmental cues. Signalling typically begins when extracellular ligands are perceived by specific receptor-co-receptor complexes at the plasma membrane, often composed of two or more RKs. While co-receptors may not always bind ligands directly, they are essential for stabilising receptor complexes and promoting their activation [5]. Ligand binding induces conformational changes that modulate the activity of the intracellular kinase domains, thereby triggering distinct downstream signalling pathways that drive finely tuned cellular responses. To ensure appropriate strength, duration and specificity, RK activity is tightly regulated by multiple post-translational mechanisms, including reversible phosphorylation, ubiquitination, dynamic receptor relocalisation and proteolytic processing [5].

Proteolytic processing, the irreversible hydrolysis of peptide bonds by proteases, modifies the structure, stability, and biological function of target proteins. This regulatory mechanism is well established in animal RKs, where proteolytic cleavage has been observed across nearly all RK subclasses, regardless of ectodomain structure. In humans, cleavage can occur extracellularly, intracellularly, or even within the membrane, and its dysregulation is linked to aberrant signalling and a range of diseases [6]. By contrast, much less is known about how proteolytic processing modulates RK function in plants [7]. In this *Pearls* article, we summarise current knowledge on RK processing in plants, highlight key challenges for future research, and draw attention to open questions regarding the mechanisms, dynamics, and regulatory principles governing this underexplored layer of receptor regulation.

## How is plant development affected by RK processing?

Hormones are central regulators of plant growth and development, with RKs often playing key roles in mediating hormone signalling. Members of the leucine-rich repeat (LRR) RK family, including Transmembrane Kinase 1 (TMK1; [8]), TMK4, and the ERECTA family (ERf), function in auxin and gibberellin signalling, respectively [9,10]. These hormones govern diverse developmental processes such as cell elongation, organ formation and tissue differentiation [11].

**Fig 1:**
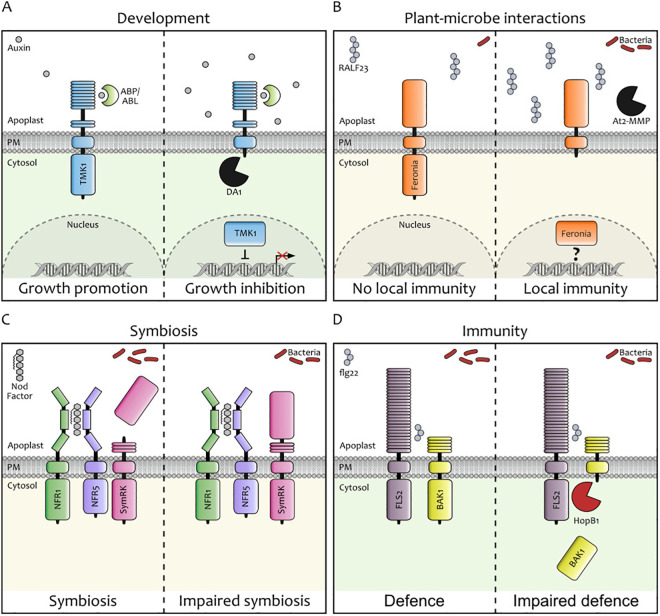
Receptor kinase processing regulates different aspects of plant life—four showcases. **A)** Auxin-triggered processing of TMK1 regulates apical hook formation. Extracellular auxin is sensed by TMK1. Elevated apoplastic auxin induces cleavage of TMK1’s cytoplasmic kinase domain by DA1-family zinc metalloproteases. The released kinase translocates to the nucleus thereby inhibiting auxin-responsive gene expression and modulating cell growth [10,13]. **B)** FER receptor processing regulates localised immunity in *Arabidopsis* roots. Increased bacterial colonisation elevates apoplastic RALF23 levels. FER senses RALF23, and high peptide levels trigger FER’s dose-dependent cleavage mediated by At2-MMP protease and nuclear translocation of FER cytoplasmic domain. This processing activates localised immunity, restricting further bacterial colonisation [17]. **C)** Cleavage of the extracellular domain of SymRK modulates symbiotic signalling. SymRK undergoes constitutive cleavage near its conserved GDPC motif, releasing the malectin-like domain (MLD) and generating a truncated membrane-bound receptor form. MLD release modulates receptor complex formation with NFR5 and promotes receptor turnover. Hampered MLD shedding impairs root nodule symbiosis and arbuscular mycorrhiza (not depicted in the figure) in *Lotus japonicus* [28,34]. **D)** Pathogen-induced cleavage of BAK1 suppresses immune signalling. During *Pseudomonas syringae* infection, the bacterial effector HopB1 is delivered into *Arabidopsis* cells, where it targets the immune receptor complex FLS2–BAK1. HopB1 cleaves the phosphorylated form of the co-receptor BAK1, thereby dampening FLS2-mediated immune signalling and promoting bacterial virulence.

Recent findings highlight proteolytic processing of RKs as a regulatory mechanism in hormone signalling. In auxin signalling, TMK1 forms a receptor complex with auxin-binding proteins (ABP/ABL) [12]. Elevated apoplastic auxin levels trigger TMK1 cleavage by the DA1 family of ubiquitin-regulated zinc metalloproteases [13], releasing its cytoplasmic kinase domain. The liberated kinase translocates to the nucleus, where it interacts with the auxin/indole-3-acetic acid (IAA) transcriptional repressors IAA32 and IAA34 to modulate gene expression and promote cell growth [10] (Fig 1A). This cleavage-dependent signalling activation is essential for apical hook formation during seedling emergence. Evidence from antibody-based detection of TMK4 in untreated plants, as well as during auxin treatment, suggest that this protein undergoes similar cleavage-based regulation as TMK1, contributing to additional auxin-mediated processes [14].

Proteolytic cleavage has also been suggested for ERf receptors, which are involved in gibberellin signalling pathways regulating traits such as inflorescence architecture and epidermal patterning, including stomata development [9]. In *Arabidopsis*, complementation of the ERf mutant *er-105* with a construct overexpressing a GFP-tagged ER allele revealed both full-length and C-terminally truncated forms of the protein [9]. However, the physiological relevance and functional role of the truncated form remains to be determined. Together, these findings underscore the emerging role of RK processing in the regulation of diverse hormone-driven developmental programs in plants.

## How are plant–microbe interactions affected by RK processing?

Plants are continuously exposed to a wide range of microbes, and RKs play a central role in detecting and responding to them, whether pathogenic, commensal, or symbiotic. Regardless of lifestyle, bacteria preferentially colonise the transition and elongation zones of plant roots [15]. To protect these vulnerable regions, plants deploy a mechanism known as localised immunity [16]. In *Arabidopsis*, the *Catharanthus roseus* RLK1‐like (CrRLK1L) receptor Feronia (FER) senses changes in bacterial abundance via accumulation of Rapid Alkalinization Factor 23 (RALF23) peptides in the apoplast [17]. Immunoblot analysis of *fer-4*/*FER-GFP* plants revealed that both bacterial exposure and RALF23 treatment induce dose-dependent cleavage of FER’s cytosolic domain. The released kinase fragment translocates to the nucleus in cells of the root transition and elongation zones, activating localised immunity, which is compromised when FER cleavage is disrupted [17]. A protease cleavage screening system, combined with *in vitro* and *in vivo* cleavage assays using mutant and overexpression lines, identified the matrix metalloprotease At2-MMP to be involved in FER cleavage. Remarkably, *At2-MMP* transcript levels increase in the root transition and elongation zones upon bacterial treatment [17]. How the activity of this extracellular protease leads to the release of the intracellular domain of FER remains an open question. Nevertheless, this study establishes a direct link between FER processing and localised immune activation in plant roots [17] (Fig 1B).

XA21, an LRR-RK originally identified in wild rice, confers resistance to the bacterial pathogen *Xanthomonas oryzae* pv. *oryzae* (*Xoo*) when introduced into cultivated rice varieties [18] and triggers immune responses upon recognition of the sulfated, *Xoo*-derived peptide RaxX [19]. Mutants of the rhomboid protease OsRBL3b, which is preferentially expressed in spikelets, exhibit elevated XA21 protein levels in this tissue compared to wild-type (WT) plants, while XA21 protein levels in leaves remain comparable to WT [20]. Although no cleavage product has been detected in rice, heterologous expression of OsRBL3b and XA21 suggest that XA21 is a substrate of OsRBL3b. An RK cleavage screen based on an alkaline phosphatase (AP)-based colorimetric assay further showed that OsRBL3b is able to cleave the transmembrane domains of several *Arabidopsis* receptors [20]. Whether OsRBL3b also targets endogenous, non-introgressed rice RKs remains unclear. These findings suggest that RK cleavage may be spatially restricted to specific organs, potentially contributing to localised regulation of signalling.

Processing of immune receptors has also been reported in response to filamentous microbes. In *Arabidopsis*, Chitin elicitor receptor kinase 1 (CERK1; [21]), a Lysin motif (LysM) RK involved in the perception of molecules from both beneficial and pathogenic microbes [22] exists as both full-length and truncated ectodomain forms, as shown by specific antibody detection under physiological conditions [23]. This truncation is not due to alternative splicing [23]. Notably, the *cerk1-4* mutant, which carries a single amino acid exchange in the second LysM of CERK1 and produces only the full-length protein, displays a hyperimmune phenotype characterised by elevated accumulation of the defence hormone salicylic acid and excessive cell death [23]. Although the responsible protease remains unidentified, these findings suggest that the cleaved CERK1 ectodomain plays a regulatory role that remains to be elucidated [23].

In addition to development and immunity, RK processing contributes to mutualistic plant–microbe interactions. Symbiosis receptor-like kinase (SymRK; [24]), a malectin-like domain leucine-rich repeat (MLD-LRR) RK, is essential for establishing root endosymbioses with nutrient-acquiring arbuscular mycorrhizal fungi and nitrogen-fixing bacteria [24–27]. SymRK undergoes constitutive proteolytic cleavage between the MLD and LRR domains near a conserved GDPC motif. This cleavage releases the extracellular MLD and generates a membrane-bound truncated fragment, SymRK-ΔMLD [28]. This processing is observed both in *Lotus japonicus* roots and upon ectopic expression in *Nicotiana benthamiana* leaves [28], suggesting that MLD shedding represents either a default state or a developmentally regulated mechanism that may modulate symbiotic signalling. In *L. japonicus*, bacterial Nod Factors are perceived by the two LysM-RKs Nod Factor Receptor 1 (NFR1, [29]) and NFR5 [29,30], initiating root nodule symbiosis-specific signalling [31,32]. While full-length SymRK interacts with both NFRs [28,33], the processed SymRK-ΔMLD fragment exhibits enhanced binding to NFR5 and outcompetes the full-length receptor [28], suggesting that MLD shedding modulates receptor complex assembly. However, SymRK-ΔMLD is unstable [28], indicating that cleavage may also promote receptor turnover. Mutations impairing MLD shedding result in defects in both arbuscular mycorrhiza and root nodule symbiosis [28,34] (Fig 1C). These findings point to extracellular receptor cleavage as a potential mechanism by which plants fine-tune their symbiotic potential.

## Can microbes manipulate plant physiology by processing plant RKs?

Plant-associated microbes have evolved sophisticated strategies to evade immune recognition, including the manipulation of RK function [35]. Among these, protease-mediated cleavage of immune RKs is emerging as a common mechanism by which diverse microbes attenuate host perception, suppress immune activation, and regulate symbiosis.

Both the bacterial pathogen *Pseudomonas syringae* and the pathogenic oomycete *Phytophthora sojae* deploy unrelated proteases to cleave the LRR-RK co-receptor BRI1-associated receptor kinase 1 (BAK1) [36,37]. BAK1 is a versatile co-receptor involved in immunity, development, and symbiosis, and its activity is tightly regulated, including through endogenous proteolytic cleavage [38]. A conserved, calcium-dependent protease cleaves BAK1 within or immediately after its transmembrane domain, and this cleavage is enhanced under biotic stress conditions. Mutation of the aspartate residue D287 renders BAK1 resistant to cleavage, and this proteolytic regulation is critical for BAK1-dependent responses [38]. In contrast to endogenous cleavage, pathogen effector-mediated cleavage in both cases results in BAK1 deactivation and reduced immune response, the cleavage sites differ: HipB1 from *P. syringae* targets the cytosolic part of the receptor, whereas PsTry from *P. sojae* cleaves the extracellular domain [39] (Fig 1D). Together, these findings highlight the diverse consequences of RK processing and underscore the strategic targeting of RK by pathogens to subvert host immunity.

Remarkably, protease deployment is not limited to pathogens. Mutualistic microbes can also manipulate host RK signalling. The broad host range rhizobium *Sinorhizobium fredii* secretes NopT, a YopT-type cysteine protease, which targets multiple RKs in *L. japonicus* [40]. NopT cleaves and deactivates the immune receptors LYK11 [41] and LYK5 [42] from *L. japonicus* and *Arabidopsis*, respectively. In addition, it interacts with the Nod Factor Receptors NFR1 and NFR5, cleaving NFR5 at its juxtamembrane domain, thus impairing rhizobial infection and nodule formation [40]. Interestingly, NFR1 can phosphorylate NopT, thereby inactivating its protease activity and providing a mechanism for host counter-regulation [40]. Together, these findings highlight microbial protease activity as a convergent and versatile strategy employed by both pathogens and mutualists to fine-tune host RK signalling and shape plant–microbe interactions.

## Conclusions and perspective

Receptor processing, defined as the irreversible proteolytic cleavage of extracellular, transmembrane, or intracellular receptor domains, is a well-characterised regulatory mechanism in mammalian RKs. By contrast, this process remains poorly understood in plants, where the analogous RKs form a substantially larger and more diverse protein family.

Recent global proteomic studies suggest that RK proteolysis might also be widespread in plants [43,44]. However, alternative explanations for the observed proteoforms such as extraction artefacts or alternative splicing have not yet been excluded. Work on individual RKs further highlights the biological challenges of studying receptor processing: artefacts may arise from ectopic overexpression or non-physiological ligand concentrations and functional readouts for cleaved fragments are in many cases still missing.

Nonetheless, emerging evidence demonstrates that RK processing:

1. significantly influences various aspects of plant development and immunity,
2. occurs broadly across monocots and dicots,
3. affects RKs regardless of ectodomain structure,
4. involves *bona fide* receptors as well as co-receptors, and
5. is hijacked by both pathogenic and mutualistic microbes.

Moreover, three studies suggest that proteolytic regulation of RKs may be spatially restricted to specific plant organs or tissues, thereby contributing to localised control of signalling [17,20,38]. Collectively, these insights position RK processing as an important, yet underexplored, regulatory layer in plant signalling.

Key open questions include:

- Does RK processing also regulate interactions with viruses, insects, or parasitic plants?
- What role, if any, does RK cleavage play in abiotic stress responses?
- How is the activity of plant or microbial proteases spatially and temporally controlled?
- Can RK processing be harnessed to improve plant breeding, stress resilience, or crop protection?

Addressing these questions will be critical for advancing our understanding of receptor dynamics in plants and for unlocking the potential of RK processing in agricultural innovation.
